# PP93 Public Consultation In Clinical Protocols: Opportunities For Stakeholder Engagement And Transparent Analysis Of Patient Contributions

**DOI:** 10.1017/S0266462325103103

**Published:** 2025-12-29

**Authors:** Nitza Muniz, Álvaro Atallah, Ana Carolina Pereira Nunes Pinto, Aline Rocha

## Abstract

**Introduction:**

Diabetes insipidus (DI) is a rare disease characterized by water balance disorders and is often associated with high morbidity. Incorporating new technologies into Brazil’s Unified Health System (SUS) requires technical rigor and public participation to ensure equitable health system. This study explored how public participation in developing a Clinical Protocol and Therapeutic Guideline (PCDT) could enhance equity and transparency in health policies.

**Methods:**

To analyze the demographic characteristics and opinions of patients and stakeholders involved in the public consultation and to describe the transparency in handling these contributions. Contributions were collected during a 90-day open public consultation and analyzed in three stages: (i) reading all submissions; (ii) categorizing key ideas; and (iii) discussing implications. This methodology provided insights into public engagement and its influence on fairness and transparency in decision-making processes, highlighting the importance of inclusive approaches to health governance.

**Results:**

A total of 15 contributions were received, with 86 percent originating from individuals, predominantly white women from southern Brazil aged 25 to 39 years. Participants rated the preliminary recommendations as very good (53%), good (40%), and average (7%). Although no suggestions were incorporated, all contributions were analyzed and responded to, with positive feedback on the protocol’s clarity. The contributions underscored the importance of a PCDT in the SUS for improving rare disease management and for fostering community engagement. The findings highlighted the need for broader knowledge dissemination to stimulate public participation and enhance diversity and inclusion across the country.

**Conclusions:**

The public consultation for the PCDT on DI emphasized the importance of transparency and community engagement in health policies. While contributions praised the protocol’s clarity, findings revealed the need to expand outreach for greater inclusivity and regional diversity. Strengthening participation processes can enhance equity, improve rare disease management, and foster more responsive and inclusive health governance in Brazil.

